# The Interaction between DNA and Three Intercalating Anthracyclines Using Electrochemical DNA Nanobiosensor Based on Metal Nanoparticles Modified Screen-Printed Electrode

**DOI:** 10.3390/mi12111337

**Published:** 2021-10-30

**Authors:** Leyla Karadurmus, Burcu Dogan-Topal, Sevinc Kurbanoglu, Afzal Shah, Sibel A. Ozkan

**Affiliations:** 1 Department of Analytical Chemistry, Faculty of Pharmacy, Ankara University, Ankara 06560, Turkey; leylakrdrms@gmail.com (L.K.); skurbanoglu@gmail.com (S.K.); 2 Department of Analytical Chemistry, Faculty of Pharmacy, Adıyaman University, Adıyaman 02040, Turkey; 3 Department of Chemistry, Quaid-i-Azam University, Islamabad 45320, Pakistan; afzals_qau@yahoo.com

**Keywords:** nanobiosensor, idarubicin, doxorubicin, epirubicin, drug–DNA interaction

## Abstract

The screen-printed electrodes have gained increasing importance due to their advantages, such as robustness, portability, and easy handling. The manuscript presents the investigation of the interaction between double-strand deoxyribonucleic acid (dsDNA) and three anthracyclines: epirubicin (EPI), idarubicin (IDA), and doxorubicin (DOX) by differential pulse voltammetry on metal nanoparticles modified by screen-printed electrodes. In order to investigate the interaction, the voltammetric signals of dsDNA electroactive bases were used as an indicator. The effect of various metal nanomaterials on the signals of guanine and adenine was evaluated. Moreover, dsDNA/PtNPs/AgNPs/SPE (platinum nanoparticles/silver nanoparticles/screen-printed electrodes) was designed for anthracyclines–dsDNA interaction studies since the layer-by-layer modification strategy of metal nanoparticles increases the surface area. Using the signal of multi-layer calf thymus (ct)-dsDNA, the within-day reproducibility results (RSD%) for guanine and adenine peak currents were found as 0.58% and 0.73%, respectively, and the between-day reproducibility results (RSD%) for guanine and adenine peak currents were found as 1.04% and 1.26%, respectively. The effect of binding time and concentration of three anthracyclines on voltammetric signals of dsDNA bases were also evaluated. The response was examined in the range of 0.3–1.3 ppm EPI, 0.1–1.0 ppm IDA and DOX concentration on dsDNA/PtNPs/AgNPs/SPE. Electrochemical studies proposed that the interaction mechanism between three anthracyclines and dsDNA was an intercalation mode.

## 1. Introduction

Chemotherapy through drug administration is an integral part of the cancer treatment program. Many compounds have been developed as potential anticancer medications. However, only a few have been proven as effective clinical therapeutic drugs. It is imperative to understand the mechanism of drug action at the cellular level to devise new potent drugs for life-threatening infections and genetic diseases. The interaction study between small molecules (drugs, organic dyes, metals) and deoxyribonucleic acid (DNA), a chief constituent in the life processes, holds great significance as a biological tool in many areas like new anticancer drugs’ screening, antitumor chemotherapy, etc. [1,2]. It is a known fact that some anticancer drugs exert their antitumorigenic activity by interacting with DNA either through cleavage of DNA nucleic acid base-pair backbone or binding, which occurs through three modes: (a) long-range electrostatic interactions, (b) noncovalent complexes by either intercalation of a planar drug molecule between the nitrogenous base pairs distorting the double-helical structure of DNA, consequently lengthening and unwinding the duplex, or (c) groove binding through intermolecular interactions which involve the interaction of crescent-shaped molecules with the DNA, resulting in inhibition of the division and transcription of cancer cells [3,4,5]. Although selective to malignant cells, many anticancer drugs have exceedingly severe side effects due to their toxicity. Hence, a fine line between their therapeutic and potentially dangerous doses exists that significantly depends on the specificity of drug metabolism and individual-based tailored therapy. Scientists are still striving to get a quick response, environmental convenience, high sensitivity, and selectivity procedures to examine biological recognition and interaction processes in bulk solutions and solid surfaces. In past years, the subject of electrochemical biosensors has been pursued relentlessly [6,7,8]. Due to their cost-effectiveness, simplicity, good selectivity, fast detection, experimental convenience, and high sensitivity compared to other conventional nucleic acid assays, such biosensors are highly significant and valuable in molecular diagnostics, drug discovery, pharmacogenomics, gene expression studies, sequencing, pathogen classification, and genotyping [9,10]. Such kinds of sensors unite the DNA layers with electrochemical transducers to generate biosensors [11]. The extremely high sensitivity of biosensors, together with their compatible nature with state-of-the-art microfabrication technologies, low cost or disposability, accuracy, minimal power requirements, and portability, renders them excellent contenders for patient diagnosis [12,13,14]. Preparing effective electrodes modified by immobilization of DNA and designing plus selectivity of probes hold immense significance in electrochemical DNA biosensors development.

The screen-printed electrodes (SPE) employed in a system provided a significant advancement by delivering cheap, reusable, stable, and dependable support to immobilize biological compounds. At present, a few SPEs based on portable electrochemical devices are available in the market for monitoring, diagnosis, and research applications [15,16]. Hence, due to their robustness, portability, and easy handling SPEs were used in this work, and surface studies were done on it by immobilizing DNA and depositing drugs on its surface.

Electrochemical methods are considered the best among other analytical techniques because of their versatility, high sensitivity, selectivity, fast and stable response, low cost, and low reagent consumption [17,18]. Subsequently, the use of chemically modified electrodes as electrochemical sensors is highly recommended [19,20,21,22]. Three strategies are used to detect anticancer drugs: (a) electrochemically by recording the oxidation signal of guanine residues in the DNA strand [23,24,25,26], (b) through an electrochemically active drug signal which is affected by DNA interactions [27,28,29,30], (c) by observing changes in the morphology of surface layer, subsequently changing surface charge transfer resistance and permeability [31,32,33].

Nanotechnology holds vast applications in the biosensor field because of its many advantages based on the tiny size of nanomaterials. Various nanoparticles are used to modify electrodes for DNA immobilization. Metallic nanoparticles are used in electroanalytical and electrocatalytic applications extensively [34]. Silver nanoparticles (AgNPs) and platinum nanoparticles (PtNPs) are significantly considered in developing electrochemical sensors and biosensors. Because of their small size, metallic nature, high surface activity, good electrical properties, and strong absorption ability, these nanoparticles act as excellent substrates to facilitate electron transfer between a broad range of electroactive species [35,36]. They have been used in many electrochemical nanosensors and biosensors designs [37,38,39,40].

**Scheme 1:**
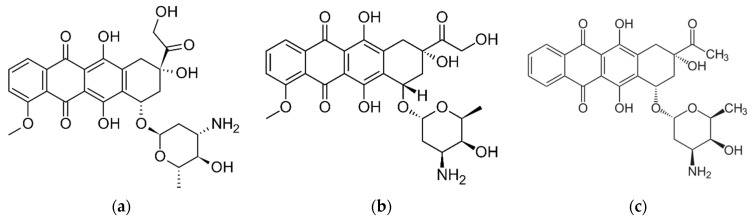
The chemical structure of (**a**) Epirubicin (EPI), (**b**) Doxorubicin (DOX), (**c**) Idarubicin (IDA).

Epirubicin (EPI), Doxorubicin (DOX), and Idarubicin (IDA) are the most widely used antineoplastic in the anthracycline class. The antitumor activity of these cancer drugs is believed to be related to their capacity to bind with DNA at a specific site by developing weak linkages with its bases, thus interfering with DNA synthesis and functioning, supporting intercalation. The anthraquinone ring in these cancer drugs intercalates between base pairs of DNA so that its long axis becomes almost perpendicular to the axis of the DNA double helix. The sugar is localized in the minor groove while one of the rings plays the role of affixer and makes the complex stable via hydrogen bonding [41,42,43,44]. In the literature survey, metallic nanoparticle-modified SPEs, have not yet been developed to monitor the interaction of the dsDNA and these anthracyclines (Scheme 1).

This paper presents the electrochemical interaction of IDA, DOX, and EPI and calf thymus double-strand DNA (ct-dsDNA) on a metallic nanoparticle-modified SPE. The electrochemical nanobiosensor was developed using a layer-by-layer modification strategy. The differential pulse voltammetry (DPV) was used to measure the response of sensitive nanobiosensor and dsDNA and these anthracyclines’ interactions based on the voltammetric signals of DNA bases.

## 2. Experimental

### 2.1. Reagents

The ct-dsDNA was purchased from Sigma-Aldrich (St. Louis, MO, USA). The ct-dsDNA stock solution was prepared in double distilled water. Its molar concentration was found out as 575 ppm using ultraviolet (UV)-visible (VIS) spectrophotometry (Agilent, Santa Clara, CA, USA). The supporting electrolyte was 0.1 M acetate buffer (AB) solution pH 4.70. The solution was kept at 4 °C for 12 h to become homogeneous. IDA, DOX, and EPI were obtained from KOCAK FARMA Pharmaceutical Company (İstanbul, Turkey). The 100 ppm stock solutions of each drug were prepared in ultra-pure water. All solutions were prepared by using analytical grade reagents and double distilled water from a Millipore Milli-Q system (Millipore, Milford, MA, USA).

### 2.2. Instrumentation

The electrochemical measurements were carried out using an IVIUM pocketSTAT electrochemical analysis system and IVIUM software package 4.1018 (Ivium Technologies, Eindhoven, The Netherlands). A screen-printed electrode (SPE) with graphite base was purchased from Dropsens^®^ (Metrohm, Oviedo, Spain). The SPE three strips electrode system contains a graphite working electrode, graphite counter electrode, and a silver reference electrode. The SPEs were activated using 0.5 M sulphuric acid (Sigma Aldrich, Darmstadt, Germany) by amperometric measurements (Ivium Technologies, Eindhoven, The Netherlands) with a current of 0.7 V and a time of 120 s. UV-Vis spectra were recorded on an Agilent Cary 60 UV-Vis Spectrophotometer (Agilent, Santa Clara, CA, USA) using a 1 cm × 1 cm quartz cell. The temperature was controlled by a Single-Cell Peltier accessory (Agilent, Santa Clara, CA, USA) with high precision. AgNPs with a size of 10 nm and PtNPs with a size of 3 nm were purchased from Sigma-Aldrich (Sigma Aldrich, Darmstadt, Germany). Operating conditions for DPV studies were a step potential of 2 mV, pulse amplitude of 50 mV, a pulse time of 10 ms, and an equilibration time of 5 s. The modified electrodes were characterized by scanning transmission electron microscopy (STEM) analysis using Zeiss Gemini 500 Field Emission SEM (30.00 kV, dropped on 300 mesh, carbon-coated copper grid) (Zeiss, Oberkochen, Germany) with an annular scanning transmission electron microscope detector.

### 2.3. Preparation of Nanosensor

PtNPs/AgNPs/SPE nanosensor was prepared via layer-by-layer deposition of nanoparticles. The different volumes of PtNPs and AgNPs nanoparticles suspensions, in the range of 1 to 10 μL, were dropped to the electrode surface. The PtNPs/AgNPs/SPE sensor was prepared by dropping the optimum volume of 1 μL PtNPs and then two-step of 5 μL AgNPs. The PtNPs/AgNPs/SPE was placed in the 0.1 M acetate buffer (AB) solution at pH 4.7, and cyclic voltammograms were recorded between the potential limits of 0.0 V and +1.5 V until a stable signal was obtained (10 cycles at a potential scan rate of 100 mV s^−1^).

### 2.4. Preparation of Nanobiosensor

The multi-layer nanobiosensor was fabricated by depositing three drops of 5 μL, each containing 50 μg mL^−1^ ct-dsDNA on the activated PtNPs/AgNPs/SPE surface. After drying the electrode at 35 °C, it was washed with AB solution at pH 4.7 for 5 s to remove loosely attached ct-dsDNA from the electrode surface. The electrode was transferred to a new AB solution at pH 4.7, followed by recording DPV under the following conditions: 50 mV pulse amplitude, 2 mV step potential, 0.07 s modulation time, 0.4 s interval time. For each experimental assay, a fresh nanobiosensor was prepared.

## 3. Results and Discussion

### 3.1. Characterization of Nanomaterials

**Figure 1:**
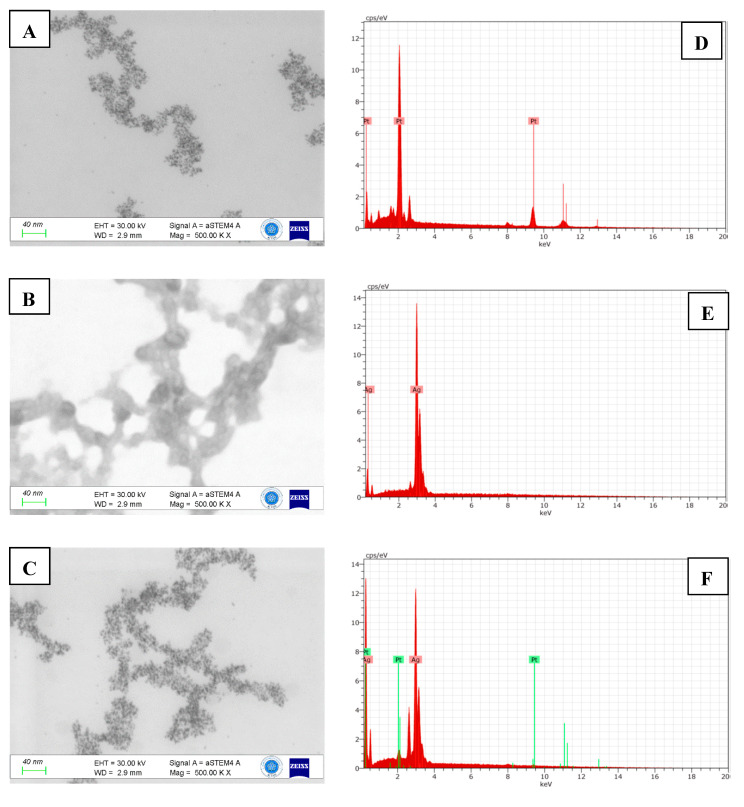
Scanning transmission electron microscopy (STEM) images of platinum nanoparticles/screen-printed electrodes (PtNPs/SPE) (**A**), silver nanoparticles (AgNPs)/SPE (**B**), PtNPs/AgNPs/SPE (**C**) with magnitude of 500.00 KX, energy dispersive X-ray analysis (EDX) analyses of PtNPs/SPE (**D**), AgNPs/SPE (**E**), PtNPs/AgNPs/SPE (**F**).

The physical appearance and surface characteristics of the SPE surface were evaluated by scanning transmission electron microscopy (STEM) and energy dispersive X-ray analysis (EDX) (Zeiss, Oberkochen, Germany). The composition of nanomaterials and surface morphologies of the PtNPs modified SPEs, AgNPs modified SPEs, and PtNPs-AgNPs modified SPEs were elucidated by STEM and EDX (Figure 1). When the STEM images of PtNPs are examined, it is clearly seen that the material has spherical morphology (Figure 1A). Since the spherical morphology increases the surface area of the material, it provides an advantage in electrochemical applications. Because the specific surface area of the material is high, it shows parallelism with the ion diffusion kinetics. When STEM images for AgNP were examined, no specific morphology could be detected (Figure 1B). This is because AgNPs have aggregation problems in the solvent media. When the STEM images for the mixture of both nanomaterials are evaluated, it is clearly seen that the PtNPs have a spherical morphology (Figure 1C). EDX analysis is an important analysis technique used to determine the elemental composition of a material. Quantitative chemical analysis of both PtNP and AgNP materials was performed via EDX analysis. EDX results for PtNP (Figure 1D) and AgNP (Figure 1E) show that the materials have specific Pt and Ag peaks, respectively, as predicted. This situation shows that the nanomaterial composition is relatively homogeneous and supports the SEM results.

### 3.2. Optimization of Modified Electrodes

The effect of metal nanoparticles on the voltammetric signals of dsDNA bases was examined. The layer-by-layer modification strategy was evaluated for preparing the nanobiosensor. The PtNPs/AgNPs/SPE sensor was prepared by dropping the optimum volume of 1 μL PtNPs and then two-step of 5 μL AgNPs.

In Table 1, the electrochemical behaviors of deoxyguanosine (dGuo) and deoxyadenosine (dAdo) were compared at dsDNA/SPE, dsDNA/PtNPs/SPE, dsDNA/AgNPs/SPE, dsDNA/PtNPs/AgNPs/SPE, and dsDNA/PtNPs/AgNPs/SPE in pH 4.70 AB by DPV.

**Figure 2:**
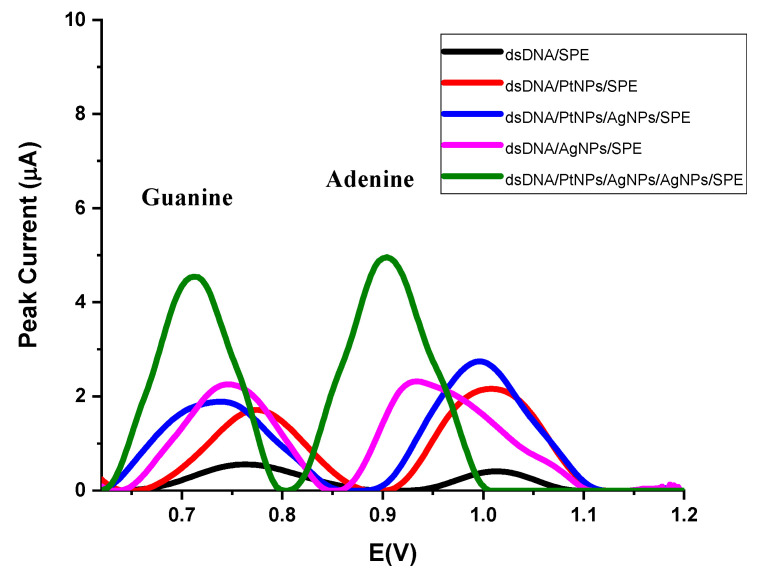
DPVs recorded at bare double-strand deoxyribonucleic acid (dsDNA)/SPE (black), dsDNA/PtNPs/SPE (red), dsDNA/AgNPs/SPE (pink), dsDNA/PtNPs/AgNPs/SPE (blue), and dsDNA/PtNPs/AgNPs/SPE (green) electrodes in pH 4.70 AB.

As seen in Figure 2, the oxidation signals of dGuo and dAdo were obtained higher at dsDNA/PtNPs/AgNPs/SPE, which was selected for further studies. On bare SPE, the peaks of dGuo and dAdo appeared at 0.764 V and 1.014 V, respectively. Compared with the PtNPs/AgNPs/SPE, the peak potentials of dsDNA bases were shifted to a less positive potential. It is concluded that the metal nanoparticles modification strategy showed the electrocatalytic effect.

**Figure 3:**
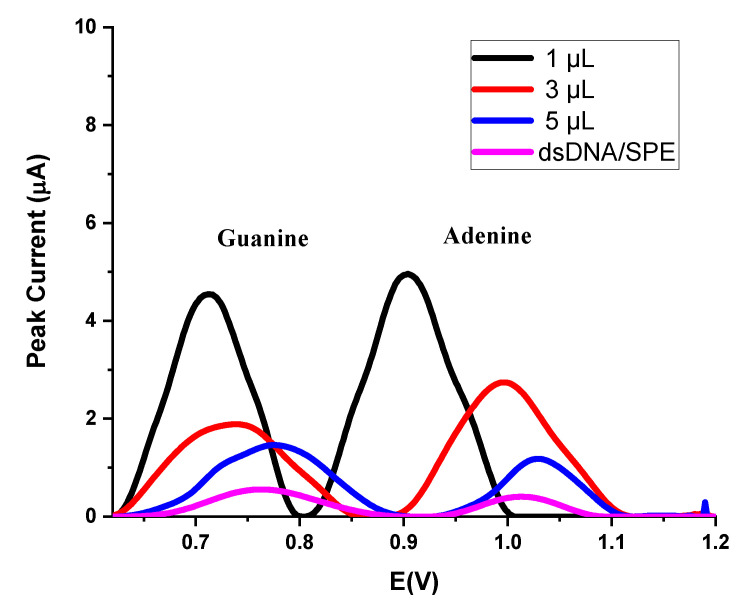
DP voltammograms at dsDNA/SPE (pink), dsDNA/PtNPs/AgNPs/SPE with different casting volume of PtNPs in pH 4.70 AB; 1 μL (black) 3 μL (red) 5 μL (blue).

The dropping volumes of PtNPs and AgNPs were varied in the range of 1–10 μL for the optimization of the nanobiosensor. As seen in Figure 3, the peak currents and potentials of dGuo and dAdo were significantly affected by the casting volume of PtNPs. The optimum value was chosen as 1 μL. As seen in Table 2, the optimum dropping volumes of AgNPs were chosen as two-step of 5 μL.

At dsDNA/PtNPs/AgNPs/SPE, the within-day reproducibility results (RSD%) for guanine and adenine peak currents were found as 0.58% and 0.73%, respectively, and the between-day reproducibility results (RSD%) for guanine and adenine peak currents were found as 1.04% and 1.26%, respectively.

### 3.3. Application of Nanobiosensor for Drugs–DNA Interaction

#### 3.3.1. The Interaction between dsDNA and EPI

**Figure 4:**
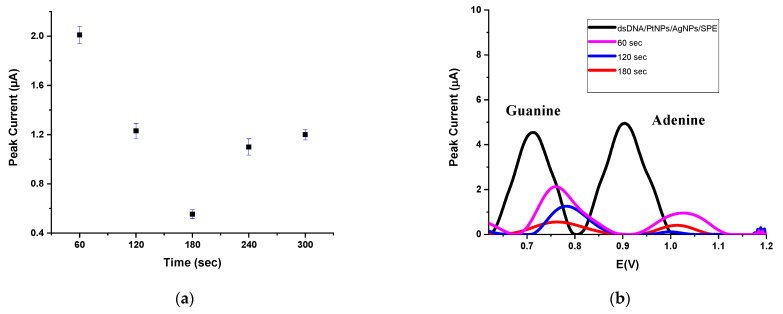
(**a**) The effect of binding time of 0.5 ppm EPI on the signal of dGuo; (**b**) DP voltammograms of dsDNA/PtNPs/AgNPs/SPE (black) with different binding time in pH 4.70 AB; 60 s (pink), 120 (blue), 180 s (red).

**Figure 5:**
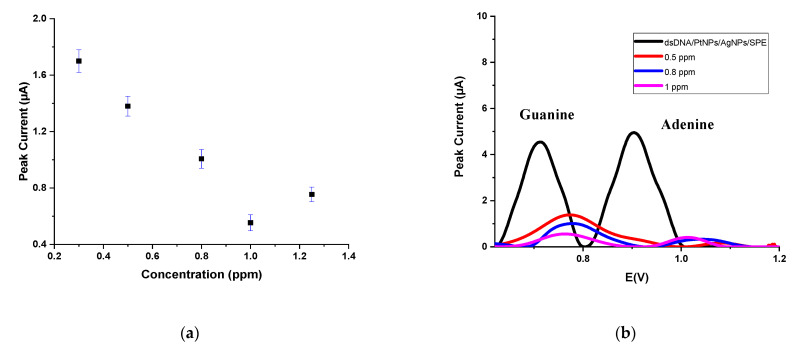
(**a**) The effect of EPI concentration on the signal of dGuo; (**b**) DP voltammograms of dsDNA/PtNPs/AgNPs/SPE (black) with different EPI concentration in pH 4.70 AB; 0.5 ppm (red), 0.8 ppm (blue), 1 ppm (pink).

EPI is an intercalating drug that may inhibit DNA and RNA synthesis [45]. The effect of binding time (Figure 4) and concentration (Figure 5) of EPI on voltammetric signals of dsDNA were evaluated by DPV on PtNPs/AgNPs/SPE. As seen in Figure 4, using dsDNA/PtNPs/AgNPs/SPE, the oxidation peaks of dGuo and dAdo were obtained at 0.764 V and 1.014 V, respectively. In addition, dsDNA/PtNPs/AgNPs/SPE was immersed into 1 ppm EPI between 1.0 and 5.0 min. Then, to remove unbound EPI molecules, the electrode was softly cleaned with ultra-pure water and then immersed into AB solution at pH 4.7 followed by recording a DPV. As seen in Figure 4a, after the interaction, the peak current of dGuo was decreased linearly until 3.0 min. Moreover, the peak current of dAdo was decreased, but this decrease was not linear (not shown). In addition, the peak potentials of dGuo and dAdo were significantly shifted to more positive potentials (Figure 4b). This shifting confirmed that the aromatic ring structure of EPI is expected to enable its intercalation into the DNA helix [42,46].

As seen in Figure 5a, the effect of EPI concentration on signals of dGuo and dAdo was evaluated in the range of 0.3–1.25 ppm EPI at the optimum binding time (3 min) using dsDNA/PtNPs/AgNPs/SPE. After interaction with EPI, the peak current of dGuo was linearly decreased in the range of 0.3–1.0 ppm EPI. As seen in Figure 5b, the peak potentials of dGuo and dAdo were shifted to more positive potentials.

#### 3.3.2. The Interaction between dsDNA and IDA

**Figure 6:**
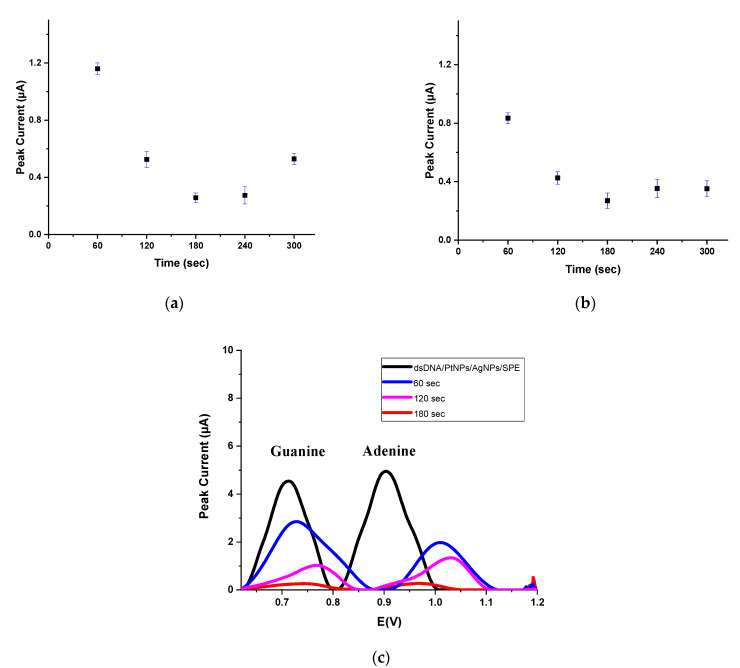
The effect of the binding time of 0.5 ppm IDA on the signal of (**a**) dGuo and (**b**) dAdo (**c**) DP voltammograms of dsDNA/PtNPs/AgNPs/SPE (black) with different binding time of IDA in pH 4.70 AB; 60 s (blue), 120 (pink), 180 s (red).

IDA is an effective drug against different cancers that inhibit cell division and DNA synthesis in cell lines with several side effects [47]. The interaction study between dsDNA and IDA is still significant concerning the suppression of cancer cell growth. To evaluate the effect of the binding time of IDA on dsDNA signals, dsDNA/PtNPs/AgNPs/SPE was immersed into 0.5 ppm IDA between 1.0 and 5.0 min (Figure 6a,b). As seen in Figure 6a,b, after the interaction, the peak currents of dGuo and dAdo were decreased linearly until 3.0 min. The peak potentials of dGuo and dAdo were shifted to more positive potentials with increasing binding time (Figure 6c). It is concluded that the aromatic ring structure of IDA is expected to enable its intercalation into the DNA helix [44,46,47].

**Figure 7:**
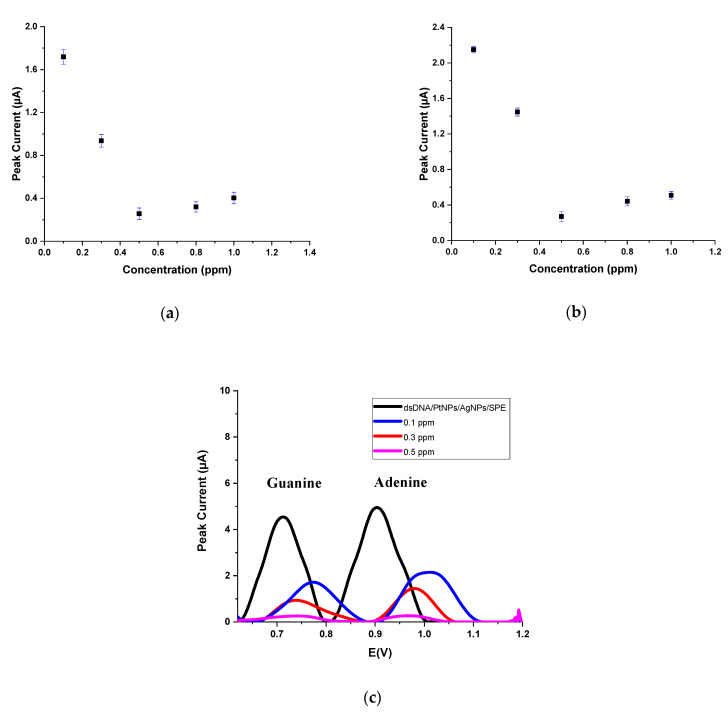
The effect of IDA concentration on the signal of (**a**) dGuo and (**b**) dAdo; and (**c**) DP voltammograms of dsDNA/PtNPs/AgNPs/SPE (black) with different IDA concentration in pH 4.70 AB; 0.1 ppm (blue), 0.3 ppm (red), 0.5 ppm (pink).

As seen in Figure 7, the effect of IDA concentration on signals of dGuo and dAdo was investigated in the range of 0.1–1.0 ppm IDA at the optimum binding time (3 min) using dsDNA/PtNPs/AgNPs/SPE. After interaction with IDA, the peak currents of dGuo and dAdo were linearly decreased until 0.5 ppm.

#### 3.3.3. The Interaction between dsDNA and DOX

**Figure 8:**
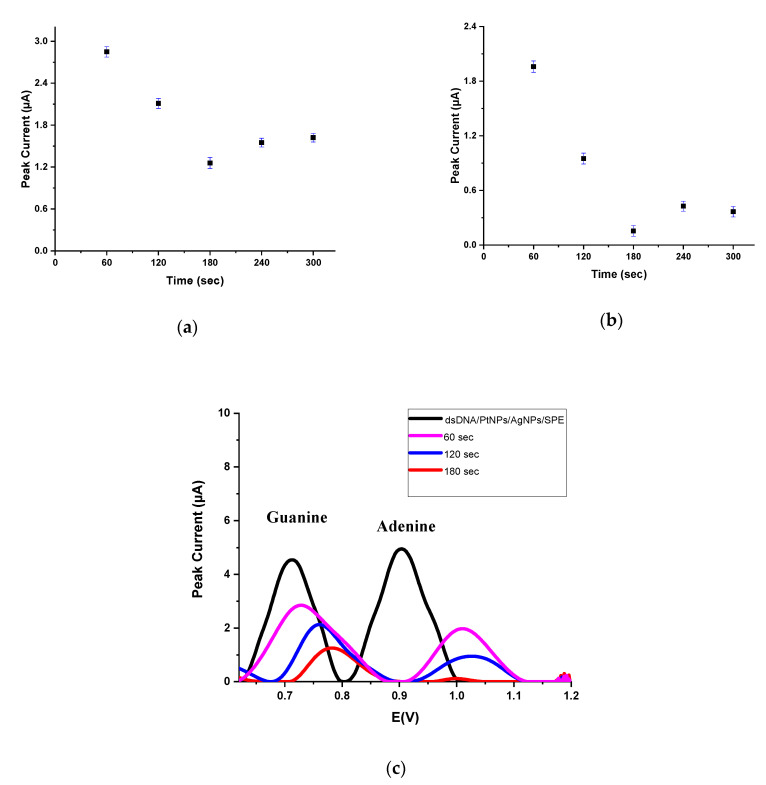
The effect of the binding time of 0.5 ppm DOX on the signals of (**a**) dGuo and (**b**) dAdo; and (**c**) DP voltammograms of dsDNA/PtNPs/AgNPs/SPE (black) with a different binding time of DOX in pH 4.70 AB; 60 s (pink), 120 (blue), 180 s (red).

The interaction between dsDNA and DOX seems to be the origin of its biological action. DOX is a well-known intercalating agent because of the insertion of its tetracyclic group into dsDNA base pairs [48]. In our study, the effect of binding time and concentration of DOX on the oxidation peaks of dGuo and dAdo were investigated by DPV using PtNPs/AgNPs/SPE. The nanobiosensor, dsDNA/PtNPs/AgNPs/SPE, was immersed into 0.5 ppm DOX between 1.0 and 5.0 min (Figure 8). As seen in Figure 8a,b, the peak currents of dGuo and dAdo were decreased linearly until 3.0 min after the interaction. In addition, the peak potentials of dGuo and dAdo were significantly shifted to more positive potentials with increasing binding time (Figure 8c). The shifting of peak potential of dGuo was linearly observed. This shifting can be explained by the intercalation of the aromatic ring structure of DOX into the DNA helix [46,48,49].

**Figure 9:**
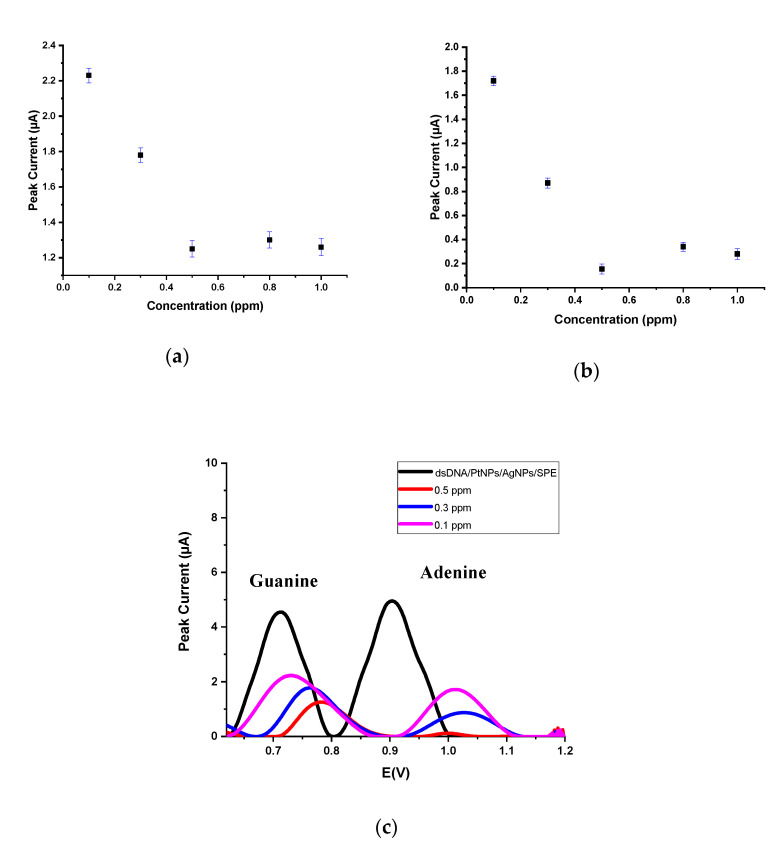
(**a**) The effect of DOX concentration on signal of (**a**) dGuo and (**b**) dAdo; and (**c**) DP voltammograms of dsDNA/PtNPs/AgNPs/SPE (dotted blue) with different DOX concentration in pH 4.70 AB; 0.1 ppm (red), 0.3 ppm (blue), 0.5 ppm (pink).

As seen in Figure 9a,b, the effect of DOX concentration on signals of dGuo and dAdo was studied in the range of 0.1–1.0 ppm DOX at an optimum binding time (3 min) on dsDNA/PtNPs/AgNPs/SPE. After interaction with DOX, the peak currents of dGuo and dAdo were linearly decreased until 0.5 ppm. As seen in Figure 9c, the peak potential of dGuo was linearly shifted to more positive potentials with increasing amounts of DOX. The peak potential of dAdo was also shifted but not linear. These results may be in accordance with the published method [48] that suggested a two-step mechanism, including the groove binding step in the A-T of the DNA region. The other step is the intercalation into the G-C of the DNA region for dsDNA-DOX interaction.

## 4. Conclusions

SPEs supply simple, sensitive, and rapid detection for the dsDNA–drugs interaction mechanism studies in electroanalytical chemistry. This interaction study would be valuable in the rational design of new DNA-targeted molecules for clinical cancer therapy. In this study, the layer-by-layer modification strategy with metal nanoparticles was conducted due to the increased surface area of SPE. The dsDNA/PtNPs/AgNPs/SPE was designed to study the anthracyclines–dsDNA binding. The proposed nanobiosensor has some advantages: single-use properties, less dsDNA solution consumption, ease of biosensor preparation, and sensitivity in comparison to published methods.

The shifting in peak potentials of dGuo and dAdo suggested the binding of EPI, IDA, and DOX with dsDNA through intercalation mode. Our electrochemical results were obtained in accordance with many published methods on the study of dsDNA–anthracyclines interaction [41,43,44,45,48]. The proposed nanobiosensor may be used as portable electrochemical devices to monitor the binding of dsDNA-intercalating agents for further clinical diagnosis studies.

## Figures and Tables

**Table 1:** The comparison of deoxyguanosine (dGuo) and deoxyadenosine (dAdo) signals in pH 4.70 acetate buffer (AB) by differential pulse voltammetry (DPV) at various modified electrodes.

	dGuo		dAdo	
Electrode	Peak Potential (V)	Peak Current (μA)	Peak Potential (V)	Peak Current (μA)
dsDNA/SPE	0.764	0.554	1.014	0.407
dsDNA/PtNPs/SPE	0.774	1.712	1.008	2.161
dsDNA/AgNPs/SPE	0.738	1.892	0.996	2.749
dsDNA/PtNPs/AgNPs (monolayer)/SPE	0.746	2.254	0.934	2.317
dsDNA/PtNPs/AgNPs (twolayer)/SPE	0.714	4.542	0.904	4.948

**Table 2:** The comparison of dGuo and dAdo signals in pH 4.70 AB by DPV at various AgNPs volume of modified electrodes.

	dGuo		dAdo	
Electrode	Peak Potential (V)	Peak Current (μA)	Peak Potential (V)	Peak Current (μA)
dsDNA/SPE	0.764	0.554	1.014	0.407
dsDNA/AgNPs (5 μL)/SPE	0.738	1.892	0.996	2.749
dsDNA/PtNPs (1 μL)/AgNPs (two-step of 3 μL)/SPE	0.728	3.387	0.934	1.852
dsDNA/PtNPs (1 μL)/AgNPs (two-step of 7 μL)/SPE	0.640	1.936	0.836	2.730
dsDNA/PtNPs (1 μL)/AgNPs (one-step of 5 μL)/SPE	0.746	2.254	0.934	2.317
dsDNA/PtNPs (1 μL)/AgNPs (two-step of 5 μL)/SPE	0.714	4.542	0.904	4.948

## Data Availability

Study did not report any external data.
